# Fermentation of Lignocellulosic Substrates Enhances the Safety and Nutritional Quality of Flake Soil for Rhinoceros Beetle Rearing

**DOI:** 10.3390/polym18010095

**Published:** 2025-12-29

**Authors:** Khanchai Danmek, Tippapha Pisithkul, Chuleui Jung, Sukjun Sun, Hyeonjeong Jang, Surat Hongsibsong, Sampat Ghosh, Ming Cheng Wu, Pichet Praphawilai, Michael Burgett, Bajaree Chuttong

**Affiliations:** 1 Biotechnology Program, School of Agriculture and Natural Resources, University of Phayao, Phayao 56000, Thailand; khanchai.da@up.ac.th; 2 Program in Biotechnology, Faculty of Science, Maejo University, Chiang Mai 50290, Thailand; tpisithkul@gmail.com; 3 Department of Plant Medicals, Gyeongkuk National University, Andong GB 36729, Republic of Korea; scv6309@naver.com (S.S.); jhj971008@naver.com (H.J.); 4 Agriculture Science and Technology Research Institute, Gyeongkuk National University, Andong 36729, Republic of Korea; 5 Research Institute for Health Science, Chiang Mai University, Chiang Mai 50200, Thailand; surat.hongsibsong@cmu.ac.th; 6 Department of Life Science, Sardar Patel University, Balaghat 481331, Madhya Pradesh, India; sampatghosh.bee@gmail.com; 7 Department of Entomology, College of Agriculture and Natural Resources, National Chung Hsing University, Taichung 402202, Taiwan; mcwu@nchu.edu.tw; 8 Office of Research Administration, Chiang Mai University, Chiang Mai 50200, Thailand; pichet.p@cmu.ac.th; 9 Meliponini and Apini Research Laboratory, Department of Entomology and Plant Pathology, Faculty of Agriculture, Chiang Mai University, Chaing Mai 50200, Thailand; michael.burgett@oregonstate.edu; 10 Department of Horticulture, Oregon State University, Corvallis, OR 97331, USA

**Keywords:** flake soil, fermentation, lignin, lignocellulose, microbial dynamics, rhinoceros beetle, waste utilization

## Abstract

The rhinoceros beetle (*Xylotrupes gideon*) requires safe and nutritious flake soil substrate for commercial rearing in northern Thailand, yet optimal lignocellulosic formulations remain undefined. This study evaluated five flake soil formulations substituting lignin-rich cadamba sawdust (0–100%) with cellulose-rich corn stover, plus cattle manure and rice bran, fermented for 90 days. Fermentation engineered the cellulose-lignin-hemicellulose matrix, reducing lignin from 25.07% to 7.30% while enriching cellulose from 29.73% to 33.83% and hemicellulose from 6.67% to 17.42%. Increasing corn stover enhanced crude protein (5.46–7.53%) and nitrogen-free extract (24.17–34.14%), creating T1 (25% substitution) as the optimal cellulose-based composite for *X. gideon* rearing. Microbial analysis showed T1-T2 supported highest α-diversity and lactic acid bacteria enrichment, suppressing pathogens like *Escherichia coli* and *Salmonella enterica*. Fermentation degraded >99% glyphosate residues (from 106 mg/kg to <0.25 mg/kg or undetectable). T1 is recommended as the optimal, sustainable flake soil for *X. gideon* rearing, balancing nutrition, microbiology, and safety while valorizing agricultural wastes.

## 1. Introduction

The rhinoceros beetle (Coleoptera: Scarabaeidae), *Xylotrupes gideon* (Linnaeus, 1767) is a species of significant economic importance, particularly in northern Thailand, where it plays a vital role in supporting cultural practices and income-generating activities. This cultural importance is prominently seen in the popular traditional game of rhinoceros beetle (locally called kwaang) fighting, which is especially prevalent in northern Thailand and involves an unusual form of partnership between man and animal [1]. Rhinoceros beetle farming is strongly dependent on flake soil, incurring substantial annual material costs and consumption. Data based on personal communication with local farmers in northern Thailand indicate a high collective economic burden: with approximately 100 farms in the region, the total annual spending on this substrate reaches 40,000 USD. This high cost is driven by the fact that an average farm of 50 rearing containers consumes 450–750 kg of substrate annually, representing a material cost of 240–400 USD per farm per year. This consumption rate translates to 9–15 kg of flake soil required per rearing container per year. These figures underscore the critical reliance on and high demand for flake soil, emphasizing the urgent need to develop a cost-effective and efficient alternative substrate to reduce production costs and support the sustainable expansion of rhinoceros beetle farming.

In addition to their economic significance, the beetles serve a vital ecological function in nitrogen fixation. Symbiotic microorganisms in their gut can convert atmospheric nitrogen into forms that are bioavailable to plants. Moreover, beetles contribute to the degradation of fungal lignocellulose, releasing nutrients sequestered in fungal biomass back into the soil [2,3,4]. This process enhances forest ecosystem fertility and supports the biodiversity of surrounding organisms [5]. Prior research indicates that compost from rhinoceros beetle larvae is essential for the decomposition of organic materials such as wood residues and plant matter, thereby directly improving soil fertility and ecosystem health [6]. Such compost significantly improves the chemical characteristics of soil, particularly by increasing soil pH and plant-available phosphorus, both of which are essential for healthy plant growth [7].

The sustainable and effective commercial production of rhinoceros beetles requires continuous research and innovation to minimize ecological impacts and reduce the depletion of natural resources [8]. The primary emphasis is on creating appropriate flake soil formulations and raising substrates sourced from hardwoods and diverse organic components [7]. For instance, wood-feeding beetles such as *Hylotrupes bajulus* (Linnaeus, 1758) and *Lyctus africanus* Lesne, 1907 exhibit significant responses to the lignocellulosic composition of the wood that they consume [9,10]. Additional studies on other xylophagous species, such as *Nicobium hirtum* (Olivier, 1790), which prefer substrates with high lignocellulose content such as dry wood, wood residues, or other dried plant materials further highlight the importance of lignocellulosic components in determining the suitability of rearing diets [11]. These findings can be directly applied to the formulation of effective flake soil for rhinoceros beetle rearing, as these beetles are similarly wood-feeding insects. Selecting substrates with an appropriate proportion of lignocellulosic materials, particularly cellulose, hemicellulose and lignin from plant sources or dried wood residues, may enhance larval growth, development, and survival rates, thereby supporting the sustainable production of rhinoceros beetles in commercial rearing systems [12,13]. The optimization of beetle rearing substrates fundamentally centers on understanding and manipulating biopolymers composition, specifically the complex between cellulose hemicellulose and lignin, which are the primary structural biopolymers in lignocellulosic plant materials [14]. Lignocellulosic substrates for beetle rearing can be naturally occurring biopolymer composites with cellulose, hemicellulose, and lignin. Fermentation processes are likely to induce not only quantitative changes in polymer composition, but also qualitative modifications in polymer structure, accessibility, and physicochemical functionality, which directly affect biological utilization [15]. The structural complexity of these biopolymers directly influences substrate properties including digestibility, microbial colonization potential, and nutrient bioavailability [16].

Commercial rearing of wood-feeding beetles, including rhinoceros beetles, both globally and in Thailand, continues to encounter substantial knowledge gaps, especially concerning the composition of rearing substrates and the formulation of appropriate flake soil. Although previous studies have provided valuable information on species biology [17], morphological traits [18,19], habitat preferences [20], and the properties of insect-derived polymers [21], research specifically addressing the nutritional requirements and essential dietary properties necessary for the development of rhinoceros beetles remains limited, including aspects such as lignocellulosic material composition, and physicochemical and biological properties of rhinoceros beetle flake soil. A study on rearing substrates for wood-feeding beetles has been conducted, with flake soil being one example as described by Ambühl et al. [8]. This substrate combines beech hardwood bark (lignin-rich), wheat bran, and water in optimized proportions and undergoes fermentation to enhance its nutritional value, promote microbial activity, and improve the stability of the rearing material. The fermentation process is essential for promoting microbial colonization and modifying the nutritional and physicochemical properties of the substrate, including pH, the carbon-to-nitrogen ratio, and water-holding capacity, all of which are key factors influencing the development of saproxylic beetles [8]. Furthermore, enriching the substrate with additional wood or other lignocellulosic material has the potential to further enhance its nutritional composition and physicochemical properties, which may significantly affect insect growth performance.

Farmers face uncertainty regarding the optimal flake soil formulation, particularly the required proportion of lignocellulosic materials. While published research is limited, personal communication with rhinoceros beetle farmers in Northern Thailand suggests that lignocellulosic materials high in lignin content significantly affect larval growth and survival. Therefore, a typical flake soil formulation consists of sawdust, cattle manure, and rice bran, which are mixed to achieve the desired moisture content and then fermented for approximately three months [22]. Moderate supplementation with lignocellulosic materials promotes larval development, whereas excessive amounts can weaken larvae and increase mortality, highlighting the need to determine the optimal substrate composition for efficient and sustainable beetle rearing. However, the use of cattle manure as a component in flake soil poses potential risks, as it is known to allow pathogenic microorganisms [23]. These risks associated with cattle manure can be mitigated by prolonged fermentation or pretreatment to reduce pathogens [24]. Despite the widespread use of fermented flake soils in commercial beetle rearing, most previous work has focused on practical recipes or simple performance outcomes, with limited quantitative analysis of how lignocellulosic composition, fermentation-driven microbiome shifts, and herbicide residues together determine substrate quality and safety [25]. Previous studies have demonstrated that fermentation can improve the performance of flake soils and other wood-based substrates for saproxylic beetles [8], but these studies have rarely integrated biopolymer composition, microbiota, and chemical contaminant dynamics within a single framework.

The present study addresses this gap by investigating flake soil formulations that combine two primary substrates in varying proportions: lignin-rich cadamba (*Neolamarckia cadamba* (Roxb.) Bosser) sawdust and cellulose- and hemicellulose-rich corn (*Zea mays* L.) stover [26]. By systematically varying the ratio of these components and characterizing each formulation in terms of lignocellulosic biopolymers, amino acid and fatty acid profiles, microbiome structure and glyphosate degradation after 90 days of fermentation, this work provides an evidence-based framework for formulating safe, nutritionally enhanced flake soils for *X. gideon* rearing under commercial-scale conditions.

## 2. Materials and Methods

### 2.1. Source of Raw Materials

The experimental flake soil formulations utilized four main components. Cadamba sawdust, serving as the primary lignocellulosic substrate, was sourced from approximately five-year-old cadamba trees. Corn stover (cellulose-rich) was obtained as a by-product from corn kernel milling for animal feed production. Cattle manure was collected locally from free-ranging native cattle *Bos indicus* L., 1758. Finally, glutinous rice (*Oryza sativa* L. var. *glutinosa* Matsum) bran was sourced from community rice mills and incorporated as an additive in experimental formulations.

### 2.2. Flake Soil Preparation and Fermentation Process

The experiment was conducted in April 2025 at a collaborating rhinoceros beetle farm, a member of the Thai Rhinoceros Beetle Conservation Association, located in Mae Kao Tom subdistrict, Mueang Chiang Rai district, Chiang Rai province, Thailand. Five experimental flake soil formulations were prepared to evaluate the effects of substituting sawdust with corn stover at various levels. The control group (C) consisted of 46.0% sawdust and 0% corn stover. The experimental groups (T1, T2, T3, and T4) systematically replaced the cadamba sawdust with corn stover at levels of 25%, 50%, 75%, and 100%, respectively. With all formulations, cattle manure (11.0%) was included as a nitrogen source, glutinous rice bran (2.0%) as a nutritional enhancement, and water (41.0%) to achieve optimal fermentation moisture content. All dry ingredients were thoroughly mixed before water was gradually added and kneaded until a homogeneous mixture was obtained in each treatment. The mixed substrates were then packed into 20 L breathable burlap sacks and fermented indoors under ambient tropical conditions typical of northern Thailand (approximately 28–30 °C, high relative humidity). The burlap sacks provided a semi-passive aeration system by allowing passive gas exchange with the surrounding air, and no mechanical turning or agitation of the substrate was performed throughout the 90-day fermentation period, allowing natural microbial activity to proceed until the flake soil was used for beetle rearing.

### 2.3. Sample Collection for Analysis

Samples were collected on Day 0 and Day 90 of the fermentation process to represent the initial and final stages. At each interval, a composite sample was created for analysis by randomly taking multiple subsamples from within each experimental container to account for potential substrate variability. Approximately 1.0 kg of the collected material was thoroughly mixed, placed in plastic bags, and stored at −40 °C to maintain integrity until analysis. This procedure ensured the analyzed samples were representative of the entire fermented substrate. For each treatment, three independent experimental containers were prepared as biological replicates (*n* = 3). At each sampling interval, a composite sample was prepared separately for each container from multiple subsamples, and all subsequent analyses were carried out in duplicate as technical replicates, with the mean of the technical replicates used for statistical analysis.

### 2.4. Characterization of Flake Soil Properties

The proximate composition of the flake soil, including crude protein, crude fat, crude fiber, ash, carbohydrate, and energy content, was determined following the procedures outlined by the Association of Official Analytical Chemists (AOAC) [27]. Amino acid profiles were analyzed using a Sykam Amino Acid Analyzer S633 (Sykam GmbH, Bayern, Germany) equipped with a Sykam LCA L-07 column, in accordance with AOAC standard protocols [28]. For sample preparation, the flake soil was hydrolyzed with 6 N HCl at 110 °C for 24 h under a nitrogen atmosphere. Prior to hydrolysis, the diets due to their high fat content underwent fat extraction using a Soxhlet apparatus with petroleum ether as the solvent. The hydrolyzed samples were then concentrated using a rotary evaporator and subsequently reconstituted in a sample dilution buffer (physiological buffer with 0.12 N citrate, pH 2.20) supplied by the manufacturer prior to amino acid analysis.

Fatty acid composition was determined using gas chromatography with flame ionization detection (GC-FID, 6890A system, Agilent, Santa Clara, CA, USA) according to the Korean Food Standard Codex (2020). Samples were derivatized into fatty acid methyl esters (FAMEs) following established protocols [29,30]. Identification and quantification were performed by comparing retention times to pure Sigma-Aldrich standards, and results are expressed as percentages of individual fatty acids in the total lipid fraction. The GC conditions included an SP-2560 column, nitrogen carrier gas (300 kPa), and an oven temperature ramped from 120 °C to 240 °C at 4 °C/min and utilizing a split ratio of 100:1.

### 2.5. DNA Sample Preparation of Flake Soil Microbiota

Metagenomic analysis was performed directly on DNA extracted from beetle diet samples without prior cultivation to preserve the original microbial community structure. DNA extraction was performed using the DNeasy PowerSoil Pro Kit (QIAGEN, Hilden, Germany), which is appropriate for complex matrices containing sawdust, such as beetle diet. A total of 250 mg of each sample was weighed into bead-containing tubes, followed by the addition of the kit-provided extraction buffer. Samples were incubated at 70 °C for 20 min and subsequently subjected to bead-beating according to the manufacturer’s protocol. The quality and quantity of extracted DNA were assessed. Qualitative evaluation was performed using agarose gel electrophoresis to assess DNA integrity. DNA concentration was measured using the Qubit dsDNA HS Assay Kit and Qubit 4 Fluorometer (Thermo Fisher Scientific, Waltham, MA, USA). DNA was extracted from three biological replicate flake soil samples per treatment (*n* = 3), and each extraction was processed once for downstream 16S rRNA sequencing.

### 2.6. 16S Metagenomic Library Preparation

Following DNA quality verification, the bacterial 16S rRNA gene was amplified using the UltraRun LongRange PCR Kit (QIAGEN). Barcoding was performed simultaneously with amplification using the Rapid Sequencing DNA—16S Barcoding Kit 24 V14 (SQK-16S114.24, Oxford Nanopore Technologies, Oxford, UK), which incorporates barcodes within the primers. All barcoded PCR products were pooled and purified using AMPure XP Beads to remove contaminants and PCR artifacts. DNA concentration was reassessed using the Qubit 4 Fluorometer to ensure appropriate input for library preparation. Sequencing adapters were ligated to DNA ends following the manufacturer’s protocol, and the necessary sequencing buffers were added to optimize pore translocation and sequencing efficiency.

### 2.7. Nanopore Sequencing and Data Analysis

Full-length 16S rRNA amplicons (V1–V9) were sequenced using the MinION Mk1d device (Oxford Nanopore Technologies). Flow cells were equilibrated to room temperature, and the storage buffer was removed prior to library loading. Sequencing was initiated and monitored in real-time using the MinKNOW™ software suite (MinKNOW for Mk1D Version 25.09.1). Raw nanopore signal data were base-called to generate nucleotide sequence files. Subsequent bacterial community profiling was conducted using EPI2ME (16s and metagenomics workflow). Reads were filtered using the workflow defaults (Q ≥ 10; full-length 16S window), and taxonomic assignment used the NCBI RefSeq targeted loci (16S) database supplied within the workflow. Across samples, per-sample depth has a median of 14,483 and a mean of 13,284, totaling 199,256 quality-filtered reads and 343 observed OTUs/features. Downstream microbial diversity, statistical analyses, and visualization were performed in MicrobiomeAnalyst2.0 (https://www.microbiomeanalyst.ca/ access on 26 November 2025).

### 2.8. Glyphosate Residues in Fermented Flake Soil

The inclusion of glyphosate in this study was to assess the chemical safety of using agricultural by-products (like corn stover) that may be contaminated with herbicide residues, and to determine the effectiveness of the 90-day fermentation process in their degradation. Glyphosate isopylammonium, a commercial herbicide product purchased from Thailand, was incorporated into five flake soil formulations (C and T1 to T4) at a concentration of 300 µg/L and subjected to fermentation for 90 days. The extraction procedure was modified according to the method described by [31]. Samples were collected at the initial stage and after fermentation for glyphosate residue analysis. Soil samples (5 g) were extracted with 0.1 M KH_2_PO_4_/Na_2_B_4_O_7_ buffer, followed by centrifugation to obtain the supernatant. Derivatization was carried out by reacting to the extract with FMOC-CI solution at 40 °C for 1 h, and the reaction was terminated with phosphoric acid. For clean-up procedure, interfering compounds were removed by liquid–liquid extraction with dichloromethane, and the aqueous phase was further purified using a C8/SAX solid-phase extraction cartridge. The analysis process was subsequently eluted with HCl-acetonitrile (1:1, *v*/*v*), filtered, and subjected to HPLC analysis.

Glyphosate analysis, based on a modified method from [32], was carried out using an Agilent 1100 HPLC system with fluorescence detection using a Discovery^®^ C18 column (25 cm × 4.6 mm, 5 µm). The mobile phase consisted of 2% phosphoric acid and acetonitrile (70:30, *v*/*v*) under isocratic conditions at 1.0 mL/min. Detection was carried out at λ_ex = 210 nm and λ_em = 315 nm. The method was validated for linearity, recovery, and precision. Calibration curves demonstrated good linearity, with r^2^ values of 0.99828 for concentrations ranging from 100–1600 µg/L (day 0) and 0.99682 for concentrations ranging from 10–160 µg/L (day 30). The precision in terms of relative standard deviation of repeatability (RSDr) was determined by analyzing three replicate samples at each fortification level for soil on the same day. The standard deviation was 0.2, and the coefficient of variation was 0.5%.

### 2.9. Statistical Analysis

The experiment was conducted using a Completely Randomized Design (CRD) to compare the effects of the five formulations under identical fermentation conditions. Each formulation was replicated three times to ensure reliable results and allow appropriate statistical analysis of differences among the treatments. The data with the normal distribution involved a one-way analysis of variance (ANOVA), followed by post hoc Tukey’s test, with a significance level set at *p* < 0.05, conducted using SPSS version 27.0 (IBM Co., Armonk, NY, USA).

## 3. Results and Discussion

Flake soils are formulated with lignocellulosic ingredients to mimic or enhance the nutritional profile of natural substrates. Commercial flake soil provides satisfactory performance for many beetle species, but their biopolymer composition and transformation during fermentation have rarely been characterized in detail. In this study, flake soils were formulated from lignocellulosic biopolymers (cadamba sawdust and corn stover) supplemented with nitrogen-rich components (cattle manure and glutinous rice bran) (Table 1). The fundamental mechanism underlying improved nutritional quality is the fermentation-driven biotransformation of structural biopolymers (cellulose, hemicellulose, lignin) into more accessible nutrient forms through the enzymatic activity of fermenting microorganisms [8]. This study compared four experimental flake soil formulations (T1–T4) and a farmer-standard formulation (C), each subjected to a 90-day fermentation period. The fermentation process employed in this study functioned as a biotechnological system for modifying lignocellulosic biopolymers. Analysis of flake soil composition (Table 1) shows a consistent reduction in lignin content across formulations from 25.07% in C to 7.30% in T4, together with increases in cellulose from 29.73% in C to 33.83% in T3 and hemicellulose from 6.76% in C to 17.42% in T4, indicating substantial shifts within the lignocellulosic biopolymer matrix. While T1 did not show a statistically significant reduction in lignin relative to the control, it followed the same directional trend and, importantly, combined moderate lignin and crude fiber levels with significantly higher nitrogen-free extract and improved microbiological stability. Although the lignin content of T1 did not differ statistically from that of the control, fermentation is known to modify lignin–carbohydrate complexes and reduce structural recalcitrance without necessarily causing a significant reduction in total lignin percentage. Therefore, the improved performance observed in T1 is likely attributable to qualitative changes in the lignocellulosic biopolymer matrix rather than to absolute lignin content alone. This pattern suggests that fermentation selectively enhanced the accessibility and bioavailability of renewable biopolymers, particularly hemicellulose and digestible carbohydrate fractions, even total lignin content did not differ significantly between C and T1. These compositional changes underpin the superior performance of T1 as a balanced flake soil formulation.

The dry matter content of the flake soils remained relatively consistent across all treatments, with no significant differences observed, and values averaging approximately 70%. Similarly, ether extract and gross energy contents were comparable among the experimental formulations, whereas crude fiber content differed significantly across the treatments. In contrast, ash content varied significantly. The highest value was observed in C, composed of high-lignin sawdust, with intermediate levels in T1 and T2, while the lowest were in T3 and T4. These results indicate that increasing the proportion of corn stover reduces the ash content of the flake soils. The flake soil formulation commonly used by farmers (C) contained approximately 5% crude protein, while the experimental formulations with increasing proportions of corn stover (T1–T4) contained approximately 6–7%, showing a significant increase in crude protein content with higher corn stover inclusion. Although reference data on the nutritional composition of this fraction of fermented flake soil are limited, Van Campenhout [33] discussed how fermentation of plant-based substrates comparable to silage can enhance digestibility and nutrient availability for insects. Experimental outcomes for parameters such as dry matter, crude fiber, ether extract, and protein are likely to follow a similar trend as observed in ensiled silage [34,35]. Nitrogen-free extract (NFE), a key indicator of soluble and digestible carbohydrates and non-fibrous components, is crucial for assessing feed quality. The NFE values increased significantly with the substitution of corn stover, resulting in the highest values in T2, T3, and T4, intermediate in T1, and lowest in C. This observation aligns with the high cellulose and energy content of corn [36], confirming that samples containing higher proportions of corn stover led to elevated NFE values.

Total crude fiber content differed significantly across treatments, with the highest levels observed in C and T1, and the lowest in T2, T3, and T4. This trend is likely due to the increased proportion of corn stover, which reduced total lignin content, thereby enhancing fermentation efficiency and influencing fiber and weight loss [34,37]. Analysis of cell wall components confirmed that the addition of corn stover increased the cellulose and hemicellulose contents while simultaneously reducing lignin proportion in the formulations [38]. These cell wall changes are consistent with the enrichment of bacteria capable of degrading cellulose and hemicellulose and producing organic acids, which stabilize pH and improve substrate safety. Rhinoceros beetles, as wood-feeding insects that primarily consume lignin-rich hardwoods [8], indicate through proximate analysis that T1 represents the most appropriate feed composition. The aligned patterns of lignocellulosic composition and dominant bacterial taxa indicate that fermentation-driven modification of cellulose, hemicellulose, and lignin is a central driver of both the chemical and microbiological quality of the flake soils. A comparative analysis with a commercial flake soil product from Taiwan, used due to the absence of a formulated product in Thailand, showed a similar nutritional profile to T1, specifically its high lignin content (22.11%) and moderate crude protein (8.17%). However, the commercial product exhibited relatively low cellulose (16.64%), hemicellulose (8.01%), ether extract (0.02%), and NFE (20.94%), indicating limited soluble energy.

In the flake soil samples, it was clearly observed that the supplementation of corn stover enhanced both the protein content and the amino acid profile of the flake soil. The average contents and standard deviations of essential amino acids (EAAs) and non-essential amino acids (NAAs) are presented in Table 2. The results indicate that valine (Val), isoleucine (Ile), leucine (Leu), threonine (Thr), histidine (His), and lysine (Lys) were the predominant EAAs, whereas glutamic acid (Glu), aspartic acid (Asp), alanine (Ala), glycine (Gly), serine (Ser), and arginine (Arg) were the dominant NAAs. Asparagine (Asn) and tryptophan (Trp) were not detected or quantified, likely due to incomplete acid hydrolysis and the inherently low concentrations of these amino acids in the sample. This observation is consistent with the findings of Ghosh and Jung [39], who reported similar results using comparable amino acid extraction and analysis methods. Although reference data are limited, available studies on amino acid profiles in plant biomass such as cottonseed show comparable levels of EAAs and NAAs which are consistent with the results obtained in this study [40]. Their study reported that amino acids were primarily distributed in the leaf blades and reproductive biomass, which are characterized by high cellulose and low lignin contents. This observation aligns well with the present findings, where the supplementation of cellulose-rich corn stover enhanced the amino acid content of the flake soil. The enhanced amino acid profiles observed with increasing corn stover incorporation represent a direct indicator of biopolymer quality transformation within the substrate. EAAs such as histidine, leucine, and valine increased significantly across treatments, reflecting both the higher intrinsic protein content of corn stover and the action of proteolytic enzymes during fermentation. This proteolysis improves the availability of proteinaceous biopolymers to rhinoceros beetle larvae, linking fermentation-driven modification of biopolymer quality to the nutritional upgrading of the flake soil [36]. From a nutritional perspective, this enrichment in essential amino acids is particularly relevant for larval growth: insects, including coleopteran larvae, generally require a set of ten essential amino acids similar to those of mammals, and optimal dietary profiles tend to mirror the amino acid composition of larval tissues. Studies on edible beetle larvae and other insect species have shown that high levels of essential amino acids such as lysine, leucine, valine, and histidine support protein deposition, cuticle formation, and overall growth performance [41,42].

The characterization and levels of fatty acids in flake soils are presented in Table 3. Although the analytical method allows for the detection of up to 37 fatty acids, only seven were identified in the samples. The fatty acid profile was dominated by palmitic, oleic, stearic, and linoleic acids, with smaller contributions from myristic, palmitoleic, and trace amounts of lauric acids, indicating a composition primarily of common saturated and unsaturated fatty acids. Overall, the lipid content of the flake soils was relatively low, which likely explains why no significant differences in fatty acid profiles were observed among the formulations. Although there is currently no information on the specific fatty acid requirements of flake soil for rearing rhinoceros beetles, the present data provide a preliminary indication that the fatty acid profile of the substrate should include at least these eight detected compounds. This observation may serve as a reference for future formulation and optimization of flake soil diets for beetle growth. The observed shifts in fatty acid saturation levels among treatments have important implications for larval physiology. Saturated fatty acids (SFAs), particularly palmitic (C16:0) and stearic (C18:0) acids, serve primarily as dense energy reserves and structural components of storage lipids, whereas monounsaturated and polyunsaturated fatty acids (MUFAs and PUFAs) contribute to membrane fluidity, cell signaling, and the biosynthesis of regulatory molecules such as eicosanoids. Experimental studies on black soldier fly, mealworm, and other edible insect larvae have shown that dietary manipulation of fatty acid profiles can influence growth rate, body composition, and stress tolerance, and that adequate provision of key PUFAs, including linoleic (C18:2 n-6) and α-linolenic (C18:3 n-3) acids, supports normal development and enhances the nutritional value of larvae as food or feed [43,44,45]. In this context, the higher relative proportions of unsaturated fatty acids observed in the corn-stover-enriched flake soils may promote membrane function and metabolic flexibility in *X. gideon* larvae, while the more saturated profiles in the farmer-standard formulation (C) likely favor energy storage but provide less potential for membrane adaptation and may reduce oxidative stability of both substrate and larval lipids.

**Figure 1:**
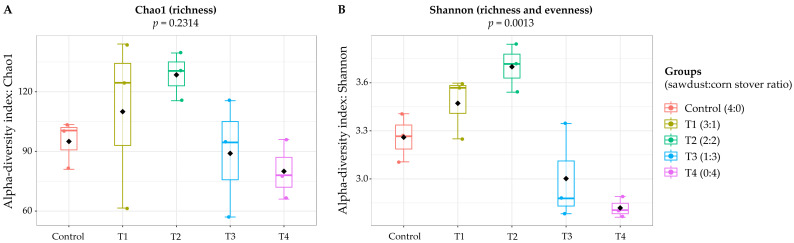
Alpha diversity of microbial communities across treatments based on richness (**A**) Chao1 index and (**B**) richness with evenness (Shannon index).

Five flake soil formulations, including C, T1, T2, T3, and T4, underwent 16S rRNA gene sequencing using the Oxford Nanopore MinION platform. The treatments (T1–T4) represented a lignin-to-cellulose gradient, with the control being most lignin-rich (sawdust-based) and T4 entirely cellulose-based (corn stover-based). Alpha-diversity analysis revealed distinct variation in microbial richness and evenness among treatments (Figure 1). Richness (Chao1 index) ranged from 77.3 ± 12.7 in T4 to 122.3 ± 18.6 in T2 (Kruskal–Wallis, *p* = 0.2314). Shannon diversity differed significantly (*p* = 0.0013), being highest in T2 (3.82 ± 0.23) and lowest in T4 (2.86 ± 0.08). Although pairwise contrasts lost significance after multiple-test correction, unadjusted comparisons indicated the greatest differences between T2 and T4 and between C and T4.

The results indicate that flake soil composition strongly influenced bacterial community diversity. Substrate formulations stemmed from a progressive replacement of sawdust with corn stover across treatments. The control, consisting primarily of sawdust and water, supported moderate bacterial diversity. T1 was associated with a slight increase in richness and diversity, suggesting that the addition of a readily fermentable insoluble carbohydrate source can stimulate microbial growth [46,47]. The most striking effect was observed in T2, where sawdust and corn stover were balanced in the mixture. This treatment yielded the highest richness and Shannon diversity, indicating that the coexistence of lignin-rich (sawdust-based) and cellulose-rich (corn stover-based) flake soils provided complementary ecological niches that supported both specialist degraders and generalist insoluble carbohydrate-utilizing bacteria. Similar observations have been reported in mixed-substrate fermentations, where diverse nutrient inputs promote microbial coexistence and functional redundancy [48,49]. The balanced substrate composition likely enhanced both richness and evenness, promoting a complex and diverse community.

In contrast, T3 and particularly T4 were associated with reduced microbial diversity. Although T3 maintained moderate richness, Shannon diversity was reduced, suggesting that higher levels of corn stover allowed dominance by a limited set of taxa. T4 (corn stover-based), which eliminated sawdust entirely, exhibited the lowest diversity across all metrics, consistent with strong community skew toward a few fast-growing carbohydrate specialists. Similar dominance effects have been noted in carbohydrate-enriched systems, where rapid fermentation by a few taxa reduces evenness and suppresses community complexity [50,51,52]. While statistical corrections limited the significance of individual pairwise contrasts, the biological trend was consistent, underscoring the role of flake soil composition in shaping microbial ecology.

**Figure 2:**
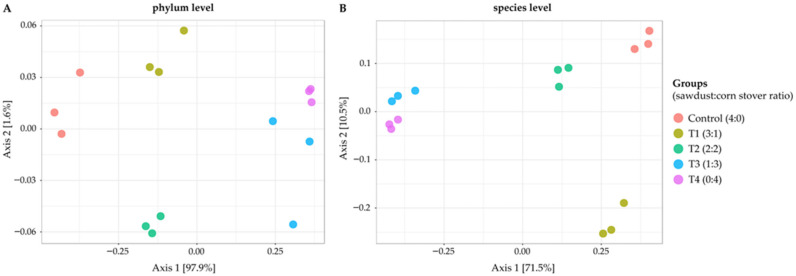
Beta diversity of microbial communities across treatments at the phylum and species levels. Principal coordinate analysis (PCoA) plots based on Bray–Curtis dissimilarity show clustering of microbial communities according to substrate composition. (**A**) At the phylum level, Axis 1 (97.9% of variation) clearly separated sawdust-based groups (control, T1) from corn stover-based groups (T3, T4), with T2 positioned intermediately. (**B**) At the species level, Axis 1 (71.5% of variation) and Axis 2 (10.5%) revealed even sharper resolution of treatment effects, with distinct clusters for each group. Control and T1 formed separate clusters, while T3 and T4 grouped tightly on the right side of the plot, indicating that microbial community composition diverged strongly as substrate composition shifted from lignocellulose-dominated to starch-rich.

Beta-diversity analysis revealed strong clustering by treatment, reflecting substrate-driven microbial structuring (Figure 2). At the phylum level (Figure 2A), principal coordinate analysis (PCoA) showed Axis 1 explained 97.9% and Axis 2 1.6% of variation. Control samples clustered tightly and distinctly from other treatments, while T1 occupied an intermediate position, and T3 and T4 (corn stover-based) clustered together on the opposite side of the ordination space. The clear separation between lignin-rich (C and T1) and cellulose-rich (T3 and T4) groups indicates a strong influence of substrate composition on community structure. At the species level (Figure 2B), Axis 1 explained 71.5% and Axis 2 10.5% of variation. Control and T1 formed distinct clusters, T2 grouped centrally, and T3–T4 clustered tightly together. The more pronounced separation at the species level indicates that treatment effects extended beyond phylum shifts to species-specific differences, confirming that substrate type fundamentally restructured microbial assemblages.

We explored the bacterial communities in commercial flake soil. In those samples, bacterial communities were predominantly composed of members of the phyla Bacillota (formerly Firmicutes) and Pseudomonadota, together accounting for more than 80% of the total microbial population. The dominance of Bacillota was notably higher in the commercial samples than in the T2 group, reflecting a community enriched in spore-forming and stress-tolerant taxa typically associated with industrially processed substrates. Minor phyla included Actinomycetota, Bacteroidota, and Myxococcota, which were present at low relative abundances (<10%).

**Figure 3:**
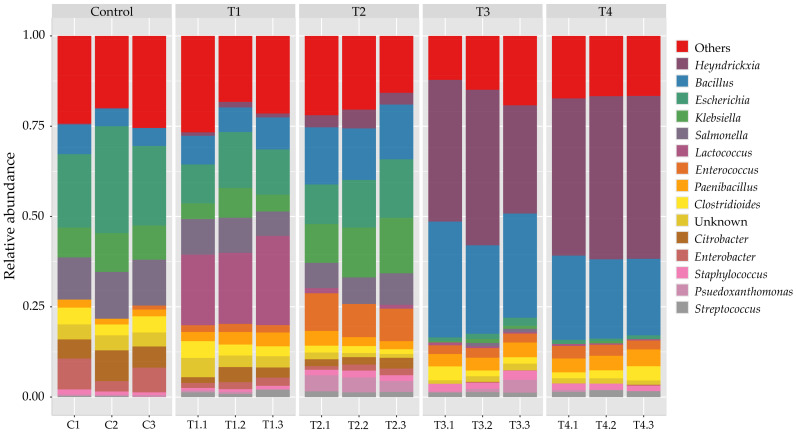
Relative abundance of bacterial communities at the genus level across treatments. Stacked bar plots illustrate the taxonomic composition of microbial communities classified at the genus rank in control, T1, T2, T3, T4, and baseline control (Day 0) samples. The dominant genera included Heyndrickxia and Bacillus, which increased in abundance particularly in T3 and T4. Other notable genera such as *Escherichia*, *Klebsiella*, *Salmonella*, *Lactococcus*, *Enterococcus*, *Paenibacillus*, and *Clostridioides* contributed to community structure at varying levels. The control and T1, T2 groups were more heterogeneous, with higher proportions of *Enterococcus* and *Lactococcus*, whereas T3 and T4 showed strong enrichment of Bacillus-affiliated taxa, indicating a treatment-driven restructuring of microbial composition.

At the genus level (Figure 3), the commercial samples were dominated by *Bacillus*,* Listeria*, and *Enterococcus*, followed by lesser proportions of *Clostridioides*,* Salmonella*, and *Escherichia*. This profile indicates a predominance of Gram-positive facultative anaerobes, suggesting that the commercial feed environment may favor endospore-forming and lactic acid bacteria (LAB) capable of withstanding dry and nutrient-variable conditions. In contrast, genera such as *Klebsiella*, *Paenibacillus*, and *Chitinophaga* were less abundant compared to T2, implying reduced representation of cellulolytic or soil-associated taxa in the commercial formulation. This suggests that the shift in substrate composition from sawdust-based (C and T1–T2) to corn stover-based (T3–T4) environments favored spore-forming and carbohydrate-utilizing taxa, thereby restructuring the stable core microbiome [53]. With moderate corn stover addition (T1–T2), the community remained Proteobacteria-rich but showed notable enrichment of *Lactococcus*, indicating that LAB were stimulated by the availability of fermentable insoluble carbohydrates [54]. This effect was particularly evident when substrates containing lignocellulose were used, as certain LAB are known to produce lignocellulolytic enzymes that function as biocatalysts, facilitating the degradation of lignocellulose in plant biomass into its constituent components [55]. This is similar to the commonly observed LAB genera involved in ensiling, including *Lactobacillus*, *Pediococcus*, *Lactococcus*, *Enterococcus*, *Streptococcus*, and *Leuconostoc*, some of which are particularly abundant in tropical grass silages [56,57]. Flake soil with high cellulose (corn stover-based; T3 and T4) but low lignin tends to undergo more efficient fermentation by LAB such as *Bacillus* and *Heyndrickxia* similar to the fermentation process observed in silage made from forage [58,59], compared to substrates with high lignin content (sawdust-based). This pattern is consistent with the ecology of Bacillaceae, which thrive in environments rich in readily fermentable plant carbohydrates. These genera are well documented for their ability to utilize soluble cellulose-derived sugars and rapidly colonize carbohydrate-rich substrates, giving them a competitive advantage when lignin, an inhibitory, recalcitrant component, is lower [49].

**Figure 4:**
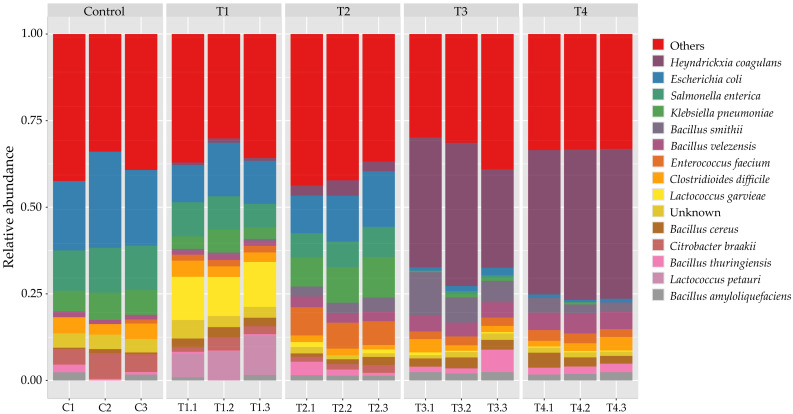
Relative abundance of bacterial communities at the species level across treatments. Stacked bar plots show the taxonomic composition of microbial communities classified at the species rank in control, T1, T2, T3, T4, and baseline control (Day 0) samples. The most dominant species observed was *H. coagulans*, which markedly increased in abundance in T3 and T4. Other species contributing to the community included *B. velezensis*, *B. smithii*, *E. coli*, *S. enterica*, *K. pneumoniae*, *E. faecium*, *L. garvieae*, and *C. difficile*. Control, T1, and T2 displayed more heterogeneous communities with higher relative abundance of opportunistic taxa such as *E. coli* and *K. pneumoniae*, whereas T3 and T4 were strongly dominated by *H. coagulans*, suggesting a treatment-associated selective enrichment at the species level.

At the species level (Figure 4), *Bacillus cereus*, *B. anthracis*, and *Listeria monocytogenes* were among the most represented identified taxa, accompanied by *C. difficile*, *K. pneumoniae*, and *E. faecium*. Although these taxa are widely distributed in environmental and feed microbiomes, their relative enrichment in the commercial product highlights the need for careful microbiological quality control, especially for substrates used in rearing biological organisms such as beetles. In the control group (C), the community was dominated by gut-associated Enterobacteriaceae, particularly *E. coli*, *S. enterica*, and *K. pneumoniae*. Opportunistic pathogens such as *C. difficile* and *E. hormaechei* were also detected, underscoring the potential risk of pathogenic reservoirs in sawdust-based flake soils. This is not surprising, as flake soil contains cattle manure, which provides a source of decomposer microorganisms and commonly harbors human pathogens, including *E. coli*, *S. enterica*, and *K. pneumoniae* [23]. With moderate corn stover supplementation (T1–T2), pathogenic Enterobacteriaceae declined, while LAB expanded significantly. These taxa are recognized for their probiotic roles in animal feed and silage fermentation, producing lactic acid that suppresses pathogens and stabilizes feed microbiota [24]. Spore-forming Bacillus species, such as *B. velezensis*, *B. thuringiensis*, and *B. amyloliquefaciens*, also increased moderately. By contrast, corn stover-based (T3–T4) were dominated by fermentative spore-formers. *Heyndrickxia coagulans* emerged as the single most dominant species. This species, formerly classified as *B. coagulans*, is notable for lactic acid production and probiotic potential in livestock feed [59,60]. Other Bacillaceae, including *B. smithii*, *B. velezensis*, *B. cereus*, and *Paenibacillus polymyxa*, were also enriched. Importantly, pathogenic taxa such as *S. enterica*, *K. pneumoniae*, and *E. coli* were nearly eliminated in T4, suggesting competitive exclusion by LAB and Bacillaceae, supported by acidification of the flake soil [24,59].

**Figure 5:**
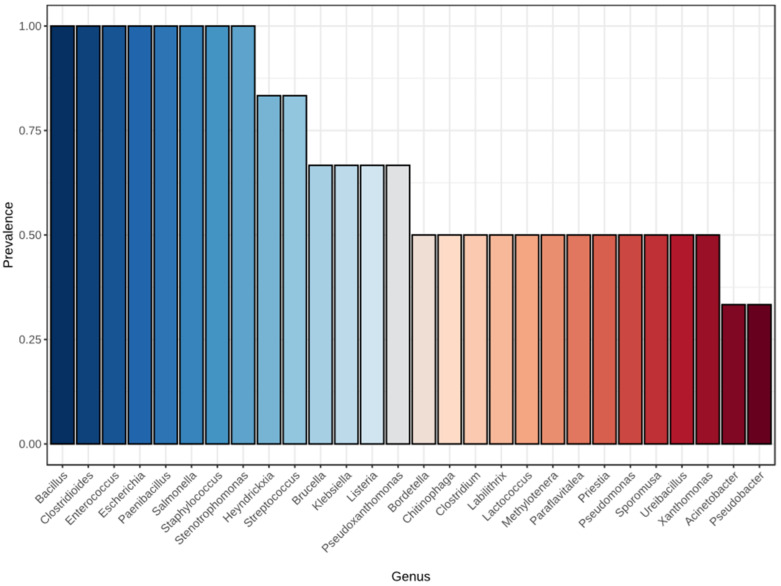
Core bacterial genera in commercial flake soil samples. Bar plot shows the prevalence of bacterial genera constituting the core microbiome of commercial flake soil across replicate samples. Genera are ordered by prevalence, defined as the proportion of samples in which each taxon was detected above the detection threshold (relative abundance ≥0.01%). Highly prevalent genera (prevalence ≥0.9), including *Bacillus*, *Clostridioides*, *Enterococcus*, *Escherichia*, and *Paenibacillus*, represent the dominant and persistent taxa within the community. Intermediate-prevalence genera such as *Klebsiella*, *Listeria*, and *Pseudoxanthomonas* occurred in 60–80% of samples, while low-prevalence genera (≤0.5) including *Lactococcus*, *Pseudomonas*, and *Acinetobacter* were sporadically detected. The gradient color scale indicates prevalence, with darker shades denoting higher occurrence frequency.

Core microbiome analysis (Figure 5) further supported the dominance of Bacillus, Listeria, and Salmonella, which exhibited the highest prevalence (>0.7) across replicates, whereas genera such as *Chitinophaga*, *Stenotrophomonas*, and *Pseudomonas* were only sporadically detected. Overall, the bacterial community in the commercial group was characterized by lower compositional diversity but higher dominance of resilient Gram-positive genera, in contrast to the more diverse and functionally varied community observed in the T2 (laboratory-prepared) group. The Bacillota enrichment observed in commercial flake soil is consistent with the ecological advantages of spore-forming taxa under desiccation, heat, and nutrient fluctuation typical of processed substrates. Endospores of *Bacillus* and *Clostridioides* withstand drying, heat, and chemical stresses due to multilayer coats, a cortex with low water content, and high dipicolinic acid, allowing these taxa to persist and dominate after industrial handling and storage [61]. In contrast, soil-associated, cellulose-degrading genera (e.g., *Paenibacillus*, *Chitinophaga*), often linked to lignocellulose turnover in natural matrices, were comparatively reduced in the commercial product, which is in line with reports of these genera as cellulolytic/xylanolytic specialists more prevalent in active plant-fiber environments rather than heat/dry-processed feeds [62].

In summary, across treatments, the dominant taxa fell broadly into two functional groups. Beneficial fermentative microbes including Bacillaceae (e.g., *Bacillus*, *Heyndrickxia*) and lactic acid bacteria (e.g., *Lactococcus*, *Enterococcus*) play well-established roles in degrading soluble plant carbohydrates, producing organic acids, and suppressing spoilage organisms during lignocellulosic fermentation. In contrast, several genera detected at low abundance (*Pseudomonas*, *Klebsiella*, *Enterobacter*) are recognized as opportunistic or potentially pathogenic, and their reduction in T2 (balanced sawdust and corn stover) suggests improved microbial stability and safety of the substrate.

Glyphosate isopylammonium was initially incorporated into all flake soil formulations (Control, T1–T4) at a nominal concentration of 300 µg/L in the aqueous phase. When residues were quantified on a dry weight basis at the beginning of the experiment (Day 0), the measured concentration was 106.06 mg/kg. The displayed value seems greater than the nominal application rate in various units; nevertheless, numerous factors account for the discrepancy. The 300 µg/L concentration in the solution differs from the analytical result per kilogram of dry substrate, as the herbicide becomes concentrated due to water absorption and loss during sample preparation and drying. Secondly, glyphosate exhibits a considerable affinity for adsorption to the mineral and organic constituents of lignocellulosic matrices, leading to an increased apparent concentration when normalized to the solid phase. Ultimately, the interactions of organic matter and pH-dependent speciation during mixing and equilibration may influence the distribution of glyphosate between liquid and solid phases.

**Figure 6:**
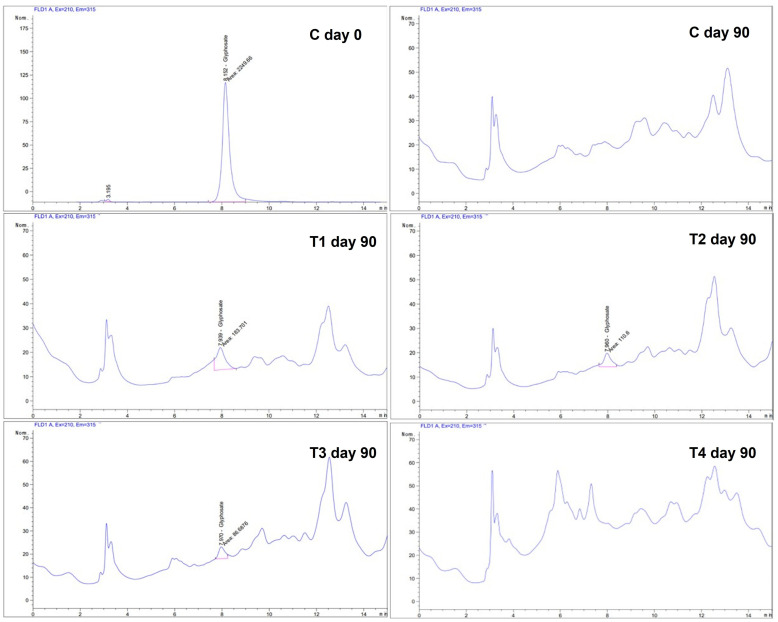
Glyphosate residue (mg/kg) in flake soil formulations: Control (C) at Day 0 and treatments (T1–T4) after 90 days of fermentation.

After the 90-day fermentation period, glyphosate residues were markedly diminished across all treatments. In the control (C0) and formulation T4, glyphosate concentrations decreased below the limit of quantification (LOQ = 10 µg/mL) of this approach and were thus undetectable. In formulations T1, T2, and T3, glyphosate residues diminished to 0.2512, 0.1465, and 0.1123 mg/kg, respectively, reflecting clearance efficiencies surpassing 99% relative to the baseline concentrations (Figure 6). The results demonstrate that the 90-day fermentation process is highly efficient in diminishing glyphosate residues in flake soils, with certain formulations achieving undetectable levels under the evaluated conditions.

Limitations: Although glyphosate residues declined to minimally detectable or non-detectable levels following 90 days of fermentation, this study did not quantify aminomethylphosphonic acid (AMPA) or other degradation products. Consequently, complete mineralization of glyphosate within the flake soil system cannot be conclusively demonstrated. The findings should be interpreted as evidence of a significant reduction in the parent compound to non-detectable levels, rather than full degradation to CO_2_, water, and inorganic phosphate. Future research should encompass targeted analyses of AMPA and other metabolites, along with mass-balance methodologies, to more precisely determine the fate of glyphosate during lignocellulosic substrate fermentation.

Although extensive research has investigated pesticide degradation in conventional silage fermentation [63,64], there are currently no studies examining glyphosate residues in flake soil systems. This represents a significant knowledge gap, as flake soil is a novel substrate with unique physicochemical properties, including high porosity, large surface area, and elevated organic matter content [8]. These properties may influence herbicide adsorption and microbial activity similar to what has been observed in silage fermentation [65]. For example, fermentation of flake soil promotes the growth of LAB including *Heyndrickxia coagulans* and *Bacillus* spp. which not only play pivotal roles in the preservation and fermentation of flake soil but may also exhibit additional functionalities, such as the potential to degrade pesticides, similar to what has been observed in silage preparation [64]. Therefore, this case study provides preliminary evidence of the potential of flake soil fermentation to mitigate glyphosate contamination and offers a foundation for future research aimed at optimizing soil formulations and microbial consortia for enhanced herbicide degradation. It is important to note that only the parent compound glyphosate was quantified in this study; key metabolites such as aminomethylphosphonic acid (AMPA) and other transformation products were not measured. The observed decreases therefore demonstrate substantial depletion of extractable glyphosate from the flake soil matrix, but do not allow conclusions about complete mineralization or the ultimate fate of glyphosate-derived phosphorus and carbon. Future studies should include AMPA and other metabolites, as well as mass-balance and mineralization measurements, to fully characterize glyphosate degradation pathways in flake soil systems.

From an applied perspective, the present results allow identification of the most suitable flake soil formulation under the conditions tested. Formulation T1, in which 25% of the cadamba sawdust was replaced by corn stover, provided the most balanced nutritional composition: crude protein and essential amino acid contents were significantly higher than in the farmer-standard formulation (C), nitrogen-free extract was increased, and lignin and crude fiber were maintained at moderate levels that preserved substrate structure and a suitable decomposition rate.

Microbiologically, T1 supported a stable community enriched in lactic acid bacteria and other fermentative taxa, with no indication of problematic overgrowth by potential pathogens, suggesting good microbial stability. Safety assessments further showed that heavy metal concentrations in T1 remained well below recognized guideline values for agricultural soils and feed or food matrices, and that glyphosate levels were substantially reduced after the 90-day fermentation period, comparable to or better than the other corn-stover-based formulations. These criteria indicate that T1 represents the most appropriate formulation for practical rhinoceros beetle rearing, offering an optimal compromise between nutritional quality, microbial stability, and chemical safety while simultaneously reducing reliance on lignin-rich sawdust.

## 4. Conclusions

This study demonstrates that lignocellulosic biopolymer composition fundamentally drives fermentation performance and substrate quality for *X. gideon* rearing. Systematic substitution of lignin-rich cadamba sawdust with cellulose-rich corn stover optimally modified the biopolymer matrix, reducing lignin, increasing hemicellulose, cellulose, crude protein, and nitrogen-free extract.

Formulation T1 (25% corn stover) achieved the optimal balance, maintaining moderate lignin (22.14%) and fiber (36.01%) for texture/decomposition while enhancing nutritional accessibility through fermentation-induced qualitative polymer modifications. T1 supported the highest microbial α-diversity with co-dominant lactic acid bacteria (*Lactobacillus*, *Pediococcus*) and lignocellulose degraders (*Bacillus*, *Clostridium*), ensuring functional stability absent in high-cellulose T3–T4 (Bacillaceae-dominated, reduced evenness).

Fermentation additionally degraded >99% glyphosate residues (106.06 mg/kg to <0.25 mg/kg or undetectable), confirming chemical safety for commercial use. T1 closely matched commercial Taiwanese flake soil (lignin 22.11%, protein 8.17%) but with superior cellulose (31.93 vs. 16.64%), hemicellulose (11.13 vs. 8.01%), and NFE (27.89 vs. 20.94%).

T1 is recommended as the optimal flake soil formulation, demonstrating how lignocellulosic biopolymer engineering via fermentation optimizes nutritional, microbiological, and safety parameters for sustainable rhinoceros beetle rearing. Limitations include analysis of parent glyphosate only (AMPA unmeasured) and lack of direct larval performance trials. Future research should validate T1 through controlled growth studies and comprehensive metabolite profiling.

However, this work has some limitations: only parent glyphosate was quantified (metabolites such as AMPA were not analyzed), and direct larval growth and long-term toxicological responses to each formulation were not assessed. Future studies should integrate controlled larval performance trials and more detailed chemical profiling of glyphosate metabolites to directly link fermentation-induced substrate characteristics with beetle growth, health, and product safety.

## Figures and Tables

**Table 1:** Ingredients and chemical composition of flake soil across treatments.

Parameter	Flake Soil Formulation					*p*-Value
Ingredient (%)	C	T1	T2	T3	T4	
Sawdust	46	34.5	23	11.5	0	-
Corn stover	0	11.5	23	34.5	46	-
Cattle manure	11	11	11	11	11	-
Rice bran	2	2	2	2	2	-
Water	41	41	41	41	41	-
**Proximate analysis (% DM)**						
Dry matter (DM)	74.31 ± 0.34	74.25 ± 1.09	72.86 ± 0.17	73.04 ± 0.10	72.98 ± 0.65	0.285
Ash	16.22 ± 1.01 ^a^	15.38 ± 0.31 ^ab^	14.74 ± 0.50 ^ab^	11.81 ± 0.46 ^c^	12.48 ± 0.52 ^c^	<0.001
Crude fiber (CF)	35.58 ± 0.84 ^a^	36.01 ± 1.53 ^a^	30.99 ± 1.19 ^b^	28.02 ± 0.58 ^b^	28.68 ± 0.87 ^b^	<0.001
Ether extract (EE)	0.90 ± 0.18	0.85 ± 0.10	0.86 ± 0.11	0.99 ± 0.18	0.99 ± 0.20	0.125
Crude protein (CP)	5.46 ± 0.27 ^b^	6.12 ± 0.28 ^ab^	6.95 ± 0.19 ^a^	7.53 ± 1.04 ^a^	7.49 ± 0.65 ^a^	<0.001
Nitrogen-free extract (NFE)	24.17 ± 2.95 ^b^	27.89 ± 1.51 ^ab^	31.72 ± 0.87 ^a^	34.12 ± 1.41 ^a^	34.14 ± 2.87 ^a^	<0.001
Cellulose	29.73 ± 2.25 ^b^	31.93 ± 0.14 ^ab^	32.61 ± 0.50 ^ab^	33.83 ± 0.70 ^a^	33.05 ± 0.22 ^ab^	<0.001
Hemicellulose	6.76 ± 3.35 ^c^	11.13 ± 1.89 ^bc^	13.18 ± 1.36 ^abc^	15.61 ± 1.93 ^ab^	17.42 ± 1.87 ^a^	<0.001
Lignin	25.07 ± 3.68 ^a^	22.14 ± 1.82 ^a^	13.60 ± 1.53 ^b^	6.67 ± 1.52 ^c^	7.30 ± 0.13 ^c^	<0.001
Gross Energy (GE) (kcal/100g)	282.6 ± 11.53	303.5 ± 35.71	314.6 ± 22.91	300.2 ± 55.01	329.9 ± 36.04	0.469

Note. C = 46:0; T1 = 34.5:11.5; T2 = 23:23; T3 = 11.5:34.5; T4 = 0:46 (cadamba sawdust:corn stover, % of total substrate; cattle manure, rice bran and water constant). The data were analyzed by ANOVA test. Mean values ± standard deviations. Means in the same row with different superscripts are significant at *p* < 0.05 level as determined by Tukey’s-b. Rows without superscript letters indicate no significant differences.

**Table 2:** Amino acid profiles (mg/100g) of flake soil across treatments.

Amino Acid (mg/g Protein)	Treatments					*p*-Value
	C	T1	T2	T3	T4	
**Essential amino acid (EAAs)**						
His	173 ± 1.5 ^e^	274 ± 1.7 ^d^	341 ± 0.5 ^c^	384 ± 1.7 ^b^	451 ± 1.1 ^a^	<0.001
Ile	277 ± 7.57 ^d^	463 ± 15.94 ^c^	456 ± 3.4 ^c^	652 ± 4.6 ^b^	722 ± 6.3 ^a^	<0.001
Leu	291 ± 4.0 ^e^	475 ± 36.2 ^d^	520 ± 5.1 ^c^	709 ± 8.0 ^b^	790 ± 1.1 ^a^	<0.001
Lys	153 ± 0 ^e^	280 ± 2.6 ^d^	305 ± 1.1 ^c^	387 ± 5.5 ^b^	448 ± 3.6 ^a^	<0.001
Met	23 ± 3.0 ^d^	31 ± 2.5 ^c^	56 ± 3.4 ^b^	55 ± 3.2 ^b^	64 ± 1.1 ^a^	<0.001
Phe	104 ± 2.5 ^c^	202 ± 2.5 ^b^	195 ± 1.5 ^b^	243 ± 82.5 ^ab^	318 ± 2.8 ^a^	<0.001
Thr	184 ± 30.0 ^c^	319 ± 5.0 ^b^	300 ± 0.5 ^b^	453 ± 8.6 ^a^	464 ± 0.0 ^a^	<0.001
Val	347 ± 4.6 ^e^	600 ± 21.0 ^c^	560 ± 11.9 ^d^	810 ± 27.7^b^	865 ± 8.6 ^a^	<0.001
**Non-essential amino acid (NAAs)**						
Ala	297 ± 2.3 ^d^	506 ± 9.2 ^c^	499 ± 1.5 ^c^	660 ± 3.7 ^b^	718 ± 0.5 ^a^	<0.001
Arg	151 ± 20.7 ^c^	298 ± 37.8 ^b^	327 ± 1.5 ^b^	482 ± 3.0 ^a^	489 ± 1.1 ^a^	<0.001
Asp	396 ± 7.0 ^e^	653 ± 9.7 ^c^	595 ± 1.1 ^d^	866 ± 9.2 ^b^	971 ± 2.8 ^a^	<0.001
Cys	ND	13 ± 6.3	9 ± 0.5	14 ± 7.5	17 ± 9.8	0.563
Gly	271 ± 1.5 ^e^	451 ± 7.0 ^c^	437 ± 2.5 ^d^	615 ± 3.6 ^b^	666 ± 0.5 ^a^	<0.001
Glu	513 ± 2.5 ^e^	883 ± 0.5 ^c^	863 ± 8.0 ^d^	1096 ± 17.0 ^b^	1260 ± 11.5 ^a^	<0.001
Pro	234 ± 13.0 ^d^	396 ± 28.0 ^c^	411 ± 6.6 ^c^	530 ± 53.0 ^b^	639 ± 1.1 ^a^	<0.001
Ser	160 ± 41.5 ^c^	298 ± 1.5 ^b^	286 ± 2.0 ^b^	419 ± 18.7 ^a^	438 ± 1.1 ^a^	<0.001
Tyr	ND	34 ± 2.3 ^d^	91 ± 8.7 ^c^	117 ± 11.2 ^b^	165 ± 2.3 ^a^	<0.001

Note. C = 46:0; T1 = 34.5:11.5; T2 = 23:23; T3 = 11.5:34.5; T4 = 0:46 (cadamba sawdust:corn stover, % of total substrate; cattle manure, rice bran and water constant). The data were analyzed by ANOVA test. Mean values ± standard deviations. Means in the same row with different superscripts are significant at *p* < 0.05 level as determined by Tukey’s-b. Rows without superscript letters indicate no significant differences. ND = not detected or below detection limit.

**Table 3:** Fatty acid profiles (mg/100g) of flake soil across treatments.

Fatty Acid (% of Total Fatty Acid)	Treatments					*p*-Value
	C	T1	T2	T3	T4	
Saturated fatty acid (SFA)						
Arachidic acid	ND	ND	ND	ND	ND	
Behenic acid	ND	ND	ND	ND	ND	
Butyric acid	ND	ND	ND	ND	ND	
Capric acid	ND	ND	ND	ND	ND	
Caproic acid	ND	ND	ND	ND	ND	
Caprylic acid	ND	ND	ND	ND	ND	
Heneicosanoic acid	ND	ND	ND	ND	ND	
Heptadecanoic acid	ND	ND	ND	ND	ND	
Lauric acid	1.95 ± 0.63	1.98 ± 0.31	2.56 ± 0.31	2.13 ± 0.14	2.50 ± 0.16	0.183
Lignoceric acid	ND	ND	ND	ND	ND	
Myristic acid	3.71 ± 0.37	4.44 ± 0.20	4.46 ± 0.62	3.70 ± 0.29	4.40 ± 0.33	0.066
Palmitic acid	56.83 ± 3.47	54.47 ± 2.79	48.98 ± 4.01	52.62 ± 7.23	53.95 ± 10.63	0.659
Pentadecanoic acid	ND	ND	ND	ND	ND	
Stearic acid	20.22 ± 1.06	21.21 ± 2.37	20.70 ± 5.30	24.23 ± 3.01	18.81 ± 0.81	0.320
Tricosanoic acid	ND	ND	ND	ND	ND	
Tridecanoic acid	ND	ND	ND	ND	ND	
Undecanoic acid (I.S)	ND	ND	ND	ND	ND	
Monounsaturated fatty acid (MUFA)						
cis-10-Heptadecenoic acid	ND	ND	ND	ND	ND	
cis-10-Pentadecenoic acid	ND	ND	ND	ND	ND	
cis11-Eicosenic acid	ND	ND	ND	ND	ND	
Elaidic acid	ND	ND	ND	ND	ND	
Erucic acid	ND	ND	ND	ND	ND	
Linolelaidic acid	ND	ND	ND	ND	ND	
Myristoleic acid	ND	ND	ND	ND	ND	
Nervonic acid	ND	ND	ND	ND	ND	
Oleic acid	34.87 ± 0.96	36.03 ± 2.96	34.38 ± 2.30	33.95 ± 2.99	34.55 ± 1.89	0.847
Palmitoleic acid	3.51 ± 0.35	5.05 ± 0.46	5.26 ± 1.10	3.45 ± 0.88	5.46 ± 0.91	0.054
Polyunsaturated fatty acid (PUFA)						
Arachidonic acid	ND	ND	ND	ND	ND	
cis-11,14,17-Eicosatrienoic acid	ND	ND	ND	ND	ND	
cis-11,14-Eicosadienoic acid	ND	ND	ND	ND	ND	
cis-13,16-Docosadienoic acid	ND	ND	ND	ND	ND	
cis-4,7,10,13,16,19-Docosahexaenoic acid	ND	ND	ND	ND	ND	
cis-5,8,11,14,17-Eicosapentaenoic acid	ND	ND	ND	ND	ND	
cis-8,11,14-Eicosatrienoic acid	ND	ND	ND	ND	ND	
Linoleic acid	13.38 ± 0.63	13.59 ± 0.71	14.40 ± 1.68	15.42 ± 1.06	15.79 ± 0.40	0.054
Linolenic acid	ND	ND	ND	ND	ND	
r-Linolenic acid	ND	ND	ND	ND	ND	

Note. C = 46:0; T1 = 34.5:11.5; T2 = 23:23; T3 = 11.5:34.5; T4 = 0:46 (cadamba sawdust:corn stover, % of total substrate; cattle manure, rice bran and water constant). The data were analyzed by ANOVA test. Mean values ± standard deviations. Means in the same row with different superscripts are significant at *p* < 0.05 level as determined by Tukey’s-b. Rows without superscript letters indicate no significant differences. ND = not detected or below detection limit.

## Data Availability

The data supporting the findings of this study are available from the corresponding author upon reasonable request.

## Abbreviations

The following abbreviations are used in this manuscript:

AOAC	Association of Official Analytical Collaboration
GC-FID	Gas chromatography with flame ionization detector
FAMEs	Fatty acid methyl esters
FMOC-Cl	9-fluorenylmethyl chloroformate
EAAs	Essential amino acid
NAAs	Non-essential amino acids
LAB	Lactic acid bacteria
